# A Pilot Randomized Controlled Trial With Home Health Aides Caring for Community-Dwelling Adults With Heart Failure

**DOI:** 10.1093/geroni/igaf122.178

**Published:** 2025-12-31

**Authors:** Madeline Sterling, Cisco Espinosa, Sasha Vergez, Joanna Ringel, Monika Safford, Jonathan Tobin, Margaret McDonald

**Affiliations:** Weill Cornell Medicine, New York, New York, United States; Albert Einstein College of Medicine, Bronx, New York, United States; VNS Health, Bronx, New York, United States; Weill Cornell Medicine, New York, New York, United States; Weill Cornell Medicine, New York, New York, United States; Clinical Directors Network, New York, New York, United States; VNS Health, New York, New York, United States

## Abstract

Home health aides and attendants (HHAs) frequently provide care to adults with heart failure (HF), but many lack training and confidence, and cannot quickly reach their nurse supervisors when they need help. In partnership with one of the largest home care agencies in New York, NY, we conducted a pilot randomized control trial (RCT) to test an education and communication-based intervention among 105 HHAs caring for HF patients. HHAs were randomized to: a) HF training (control) or b) HF training plus mHealth app that allowed HHAs to message nurses in real-time (intervention) and followed for 90 days. Main outcomes: 1) feasibility 2) acceptability and 3) effectiveness (primary outcomes: HF knowledge Caregiver Self-Efficacy (SE), secondary outcome: self-reported preventable 911calls). Data on patient outcomes (ED visits, hospitalizations) were collected. Mixed effects models were used to compare trajectory of all outcomes between and within study arms. Qualitative data was analyzed thematically. The presentation will focus on the findings of the RCT, including completion and retention rates, feasibility and acceptability of the intervention, and HHA and patient outcomes. Overall, we found that training improved HHAs’ HF knowledge and caregiving SE, with the largest gains among those with lowest baseline scores. The chat feature (intervention) did not significantly improve primary outcomes but did reduce preventable 911 calls (secondary outcome). Fewer ED visits occurred among patients cared for by intervention arm HHAs. The findings can inform the design of a future large-scale trial, and how to better support and integrate HHAs providing care HF care.

